# The Association Between Fear of Coronavirus Disease 2019, Mental Health, and Turnover Intention Among Quarantine Hotel Employees in China

**DOI:** 10.3389/fpubh.2021.668774

**Published:** 2021-05-31

**Authors:** Yi-Man Teng, Kun-Shan Wu, Dan Xu

**Affiliations:** ^1^College of Modern Management, Yango University, Fuzhou, China; ^2^Department of Business Administration, Tamkang University, New Taipei, Taiwan; ^3^College of Modern Management, Yango University, Fuzhou, China

**Keywords:** COVID-19, quarantine hotel, mental health, fear of COVID-19, turnover intention

## Abstract

During the coronavirus disease 2019 (COVID-19) pandemic, quarantine hotel employees face a higher risk of infection while they host quarantine guests from overseas. This is the first research to empirically investigate the psychological effects of operating a quarantine hotel on its employees. The empirical results indicate that heightened fear of COVID-19 leads to adverse mental health issues for quarantine hotel employees and confirm that depression, anxiety, and stress have a significant influence on turnover intention. These findings contribute to the extant knowledge base by uncovering the role of mental health in employee turnover intention. Based on the results, implications are presented for practitioners.

## Introduction

The coronavirus disease 2019 (COVID-19) pandemic has had a significant impact on the health and safety of each country's population, as well as ongoing effects to their economies and societies (1). The pandemic paused the majority of hospitality and tourism industry activities, causing a rapid decline in the average occupancy rates of hotel accommodation (2). The catastrophic result of the COVID-19 crisis is that millions of hospitality industry workers are now unemployed (3).

Since the COVID-19 outbreak, the Chinese government has taken measures to prevent the epidemic from further exacerbation (4), while each country has implemented new policies and procedures in an attempt to control the pandemic. Common measures include social distancing, lockdowns, and directives for the general public to remain in their homes (5, 6). In addition, governments worldwide have enforced strict safeguard measures and quarantine restrictions on all passengers from overseas arriving in or returning to the country, including a compulsory 14-day quarantine period in a designated hotel. Many governments have expropriated hotels to be used as temporary quarantine accommodation, referred to as the “quarantine hotel (QH).” Following the view of Kolkova (7), companies are responding to this need by intensifying accuracy requirements for forecasting market demand. The QH is the hotel industry's resilience strategy in response to a major global crisis.

The world is still managing the COVID-19 pandemic, and while global attention is largely focused on the impact to physical health, its effect on mental health cannot be ignored (8). Health authorities have recognized that COVID-19 can worsen mental health conditions (9). A health crisis, such as the pandemic, can lead to psychological changes that can be instigated by fear, anxiety, depression, or insecurity (10). Prior articles show that in China, 24.9% of college students suffer from anxiety toward COVID-19 (11), and 37.5% of Chinese international students' post-traumatic stress disorder (PTSD) PCL-C scores measure as moderate or severe (12). In addition, recent evidence reveals that 35% of China's general population are psychologically distressed (13) and experience considerable stress, anxiety, and depression (14), with similar findings evident among the people of Saudi Arabia, Russian-speaking respondents, and Israeli University students (15, 16). Some tourism literatures also evidence a psychological impact on tourists and the hotel industry throughout the COVID-19 pandemic (17–19). Thus, there is an urgent need to understand the effects of the COVID-19 pandemic on mental health, as the current crisis could cause panic (20).

Fear has been one of the primary emotional responses throughout the pandemic (21, 22). As understanding of COVID-19's biology remains limited, fear regarding the uncertainty of its transmission has increased (15). Prior studies evidence that throughout a pandemic, people tend to experience fear of infection, which results in stress, depression, and anxiety (23), and that uninfected people have a fear of contact with confirmed COVID-19 patients. This is an irrational response that may result from increased fear of COVID-19 (5, 24).

Panic and fear can create further issues for positively diagnosed COVID-19 patients and their families as they may feel socially excluded. This can increase their risk of developing psychological problems such as depression and adjustment disorders (8, 10). Other fears may stem from concerns regarding work-related changes and retaining employment. Recent research also reveals that COVID-19 influences mental health outcomes such as anger, anxiety, boredom, depression, fear, stigma, stress, and PTSD (6, 25–30). Globalization and increased access to information have made it easy for concerns related to uncertainty to negatively shift toward fear and anxiety, causing more psychological distress among the general public (24, 31).

Currently, in China, the COVID-19 pandemic is well-controlled; however, confirmed cases from overseas continue to increase. During the pandemic, QH employees are exposed to a higher risk of infection while they host quarantine guests from overseas. The role of a QH worker resembles that of a medical or health-care professional, as they both have direct contact with quarantine guests. As a result, they are more vulnerable to COVID-19's main route of transmission, which is respiratory droplets. The dilemma for QH employees is how to manage the additional duties of working in a QH, combined with the greater chance of experiencing psychological issues (depression, anxiety, and stress), and the higher risk of infection for themselves and their families and friends as a result of their increased exposure to COVID-19. Also, QH employees may not have paid sick leave, so they could be at risk of losing their job if they need to self-quarantine or care for an infected family member (32).

Worldwide, human resources are paramount when trying to achieve a competitive advantage in business (33), the loss of which can potentially threaten a hotel's existence. QH employees are affected not only physically by the impact of COVID-19 to their daily duties but also psychologically by the increased risk of contracting the virus, creating fear and persistent mental health challenges such as depression, anxiety, and obsessive behavior (34). Working closely with COVID-19 has also increased their perceived risk, psychological strain, and feelings of helplessness, which has led to some QH employees questioning whether to continue their careers in the hotel industry. Fear, a negative emotion, and the poor mental health of QH employees as a result of COVID-19 may cause an increase in turnover intention.

Even as this article is being written, the COVID-19 pandemic continues to evolve and spread. As empirical investigation on the mental health response to COVID-19 among QH employees remains scarce, this research intends to explore the association between fear of COVID-19, mental health, and turnover intention of QH employees in China.

## Materials and Methods

### Association Between Fear of Coronavirus Disease 2019 and Mental Health

Fear is an instantaneous feeling that occurs in response to the awareness of a threat (26, 35) and is one of the major underlying factors that can lead to physical and mental health issues (36). A prior study evidenced that there is a profound psychosocial impact on people at an individual level during outbreaks of infection, where they are more likely to experience a fear of falling sick or dying (23). Assessing fear during the ongoing COVID-19 pandemic is essential, and understanding its impact on mental health is critical (20).

Literature from Saudi Arabia and Turkey discuss the association between fear of COVID-19 and mental health in the general population and health-care workers during an epidemic. They state that fear of COVID-19 is a significant contributor to mental health problems, such as intolerance of uncertainty, depression, anxiety, and stress (37–40). Furthermore, Bakioglu et al. (26) indicated a positive association between fear of COVID-19 and depression, anxiety, and stress in the general population of Turkey.

Khattak et al. (22) discussed how fear of the COVID-19 pandemic impacted the mental health of nurses in Pakistan, and they found that it had a significant affect. After adapting the Fear of COVID-19 Scale (FCV-19S) into Turkish, Satici et al. (8) proved that fear of COVID-19 increased mental health issues and decreased life satisfaction. Furthermore, Harper et al. (41) and Taylor et al. (42) demonstrated that fear of COVID-19 had a significant influence on depression and anxiety. Based on these insights, this study's first hypothesis is:

H1. Fear of COVID-19 is positively associated with mental health status, specifically depression, anxiety, and stress.

### Association Between Mental Health and Turnover Intention

Turnover intention is an employee's intention to look for a new job and leave their current place of employment (43). An increase in staff turnover intention can have serious consequences for an organization (44), as employees are a crucial asset in gaining a competitive advantage (45).

The negative effects of voluntary turnover intention are often discussed in the hospitality industry (44, 46). Previous literatures state that depressed hotel employees have a greater intention to leave their jobs (47, 48). Some researchers also evidence that employees with more stressful roles, such as hotel employees and nurses, will have increased turnover intention (49, 50). QH employees have to cope with not only the physical demands of their new roles but also the threat of contracting COVID-19 and its potential psychological effects. Due to the continuous threat of infection, depression, anxiety, and stress levels in QH employees have increased and could potentially cause a rise in turnover intention. Based on the abovementioned literatures, the second hypothesis is:

H2. Mental health, specifically depression, anxiety, and stress, is positively related to turnover intention.

### Association Between Fear of Coronavirus Disease 2019 and Turnover Intention

Previous studies on nurses found that their fear of COVID-19 and the increased risk of infection strengthened their intentions to leave nursing and seek alternative employment (51). Thus, nurses with higher levels of fear and anxiety also have increased turnover intention (52). A study on Pakistani nurses revealed that their perceived threat of COVID-19 leads to higher levels of anxiety and turnover intention (53). Moreover, De los Santos and Labrague (21) also showed that heightened fear of COVID-19 enhanced mental health issues and, consequently, turnover intention, and decreased nurses' job satisfaction. A recent survey suggested that the COVID-19 crisis has instigated fear and an increase in mental health challenges among hospitality workers (34). According to the abovementioned literatures, the third hypothesis is:

H3. Fear of COVID-19 is positively associated with turnover intention.

**Figure 1:**
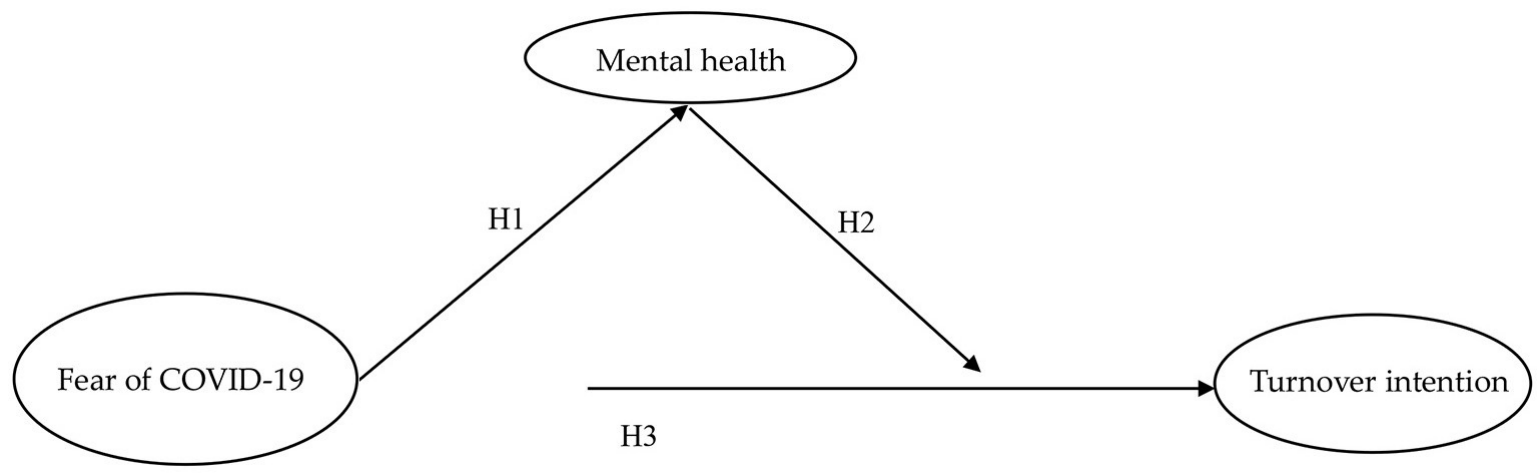
Research conceptual model. Source: own research.

With reference to the previous literatures, the conceptual model proposed in this study is presented in Figure 1.

### Questionnaire Design and Measurement Scales

The questionnaire consisted of two sections. The first section outlined the demographic variables, including gender, age, education, and monthly income. The second section included three scales for fear of COVID-19, mental health effects (depression, anxiety, and stress) caused by COVID-19 and COVID-19-related turnover intention.

The seven-item scale created by Ahorsu et al. (24) measured fear of the COVID-19 pandemic. Each item was scored using a self-rated Likert scale from 1 to 5. The Chinese version of the Depression Anxiety Stress scale (DASS-21) based on Dong et al. (54) was adapted to measure the mental health status of QH employees in China. The DASS-21 evaluates the mental health of respondents using the three subscales of depression, anxiety, and stress, each containing seven items (55). All items were scored using a 4-point Likert scale ranging from *0* (*didn't apply to me at all*) to *3* (*much or mostly applied to me*). Three items based on Vigoda (56) were adapted to assess the impact of COVID-19 on the turnover intention of QH employees. The 5-point Likert scale was employed to rate the responses, which ranged from *1* (*strongly disagree*) to 5 (*strongly agree*).

### Data Collection and Procedure

The data were collected using the convenience sampling method from QHs in Xiamen, Fujian Province, China. There are approximately 50 QHs in Xiamen, as it is the only city in the Fujian Province with airports receiving international flights. Participants were recruited from May 20 to June 10, 2020. The human resource managers from the selected QHs used their staff WeChat group (similar to WhatsApp) to post a recruitment advert. This advert invited employees to participate in the study and explained its background, procedures, and purpose. The survey was conducted with the agreement of the QH employees, and all their personal information was kept confidential and anonymous.

### Methods of Analysis

The partial least-squares (PLS) model was used for data analysis in this study. According to Pavlou and Fygenson (57), PLS places minimum restrictions on measurement scales, sample size, and residual distributions. To assess the research framework, PLS was conducted in two stages. First, it tested the reliability (indicator and internal consistency reliability) and validity (convergent and discriminant validity) of the measurement model. Second, it also evaluated the structural model and tested the hypotheses (58).

## Results

### Respondent Demographics

A total of 170 participants answered the questionnaire, 58.2% of whom were women. In terms of ages, 28.2% were Gen X (born 1965–1976), 53% were millennials (born 1977–1995), and 18.8% were Gen Z (born 1996+). With regard to education levels, 11.8% of employees had *middle school or below degree*, 27.6% had *senior high school/vocational school*, 33.5% had *junior college*, and the remaining 27.1% had an *undergraduate or above degree*. Three quarters (75.3%) of the employees indicated that their individual income was lower than 6,000 RMB/month, 17.1% indicated their individual income was higher than 6,000 RMB/month, and the remaining 7.6% did not want to talk about their individual income.

The prevalence of depression, anxiety, and stress (DAS) in QH employees is shown in Table 1. Using a cutoff score of 14 for depression, 10 for anxiety, and 19 for stress, 43.5% of respondents reported symptoms of depression, 68.2% symptoms of anxiety, and 8.2% symptoms of stress. These results demonstrate that among the respondents, depression symptoms, measured as “Yes/No” responses (Y/N), are 43.5/56.5 = 0.769 (ns); anxiety symptoms Y/N 68.2/31.8 = 2.144 (significant); and stress symptoms Y/N 8.2/91.8 = 0.09 (ns). The prevalence of anxiety is 2.1 times more in this sample and confirms that most QH employees in China have severe symptoms of anxiety, the most serious of the three mental health issues (depression, anxiety, and stress). QH employees' anxiety likely results from concerns regarding the risk of infection while completing their duties and/or excessive exposure to negative media coverage of the COVID-19 pandemic.

**Table 1:** Prevalence of DAS in QH employees in China.

**Characteristics**	**Response**	**Numbers (%)**
Depression symptoms	No (<14)	96 (56.5)
	Yes (≥14)	74 (43.5)
Anxiety symptoms	No (<10)	54 (31.8)
	Yes (≥10)	116 (68.2)
Stress symptoms	No (<19)	156 (91.8)
	Yes (≥19)	14 (8.2)

*DAS, depression, anxiety, and stress; QH, quarantine hotel*.

As indicated in Table 2, the different fear levels of COVID-19 were insignificant between genders. The one-way ANOVA test showed a significantly lower total score on the FCV-19S in *undergraduate and above* QH employees than in *senior high school/vocational school below* employees (*F* = 2.573, *p* < 0.1). Additionally, QH employees with monthly incomes of 6,000 RMB/month or below had a higher total FCV-19S score than those who did not want to talk about their income (*F* = 2.47, *p* < 0.1).

**Table 2:** Correlations between key study variables.

**Characteristic**		**Fear score (mean ± SD)**	**t/F**	**Depression score (mean ± SD)**	**t/F**	**Anxiety score (mean ± SD)**	**t/F**	**Stress score (mean ± SD)**	**t/F**	**Turnover intention score (mean ± SD)**	**t/F**
Gender	Male	22.55 ± 7.77	0.022	5.52 ± 4.22	1.248	5.90 ± 4.30	1.515	6.28 ± 4.50	1.222	6.11 ± 2.93	0.609
	Female	22.53 ± 6.41		4.84 ± 2.92		5.05 ± 3.02		5.57 ± 3.15		5.87 ± 2.30	
Age	Gen Z	21.47 ± 8.21	0.485	5.12 ± 4.35	0.418	5.28 ± 4.18	1.695	5.66 ± 4.37	1.298	6.72 ± 3.10	1.942
	Millennials	22.68 ± 7.25		4.92 ± 3.71		5.03 ± 3.83		5.54 ± 4.06		5.91 ± 2.69	
	Gen X	22.95 ± 5.55		5.50 ± 2.42		6.21 ± 2.64		6.60 ± 2.58		5.58 ± 1.80	
Education level	Middle school and below^a^	24.80 ± 8.40	**2.573^#^ (d** **<** **a,b)**	5.25 ± 2.94	3.463^*^ **(b** **>** **c,d)**	6.60 ± 3.59	6.183^**^ **(b** **>** **c,d)**	6.55 ± 3.78	4.694^**^ **(b** **>** **c,d)**	5.65 ± 2.89	1.393
	Senior high school/vocational school^b^	23.74 ± 6.49		6.45 ± 3.49		6.91 ± 3.46		7.38 ± 3.50		6.36 ± 2.15	
	Junior college^c^	22.40 ± 6.28		4.54 ± 3.73		4.54 ± 3.71		5.09 ± 3.89		5.49 ± 2.61	
	Undergraduate and above^d^	20.48 ± 7.31		4.43 ± 3.24		4.41 ± 3.10		4.98 ± 3.45		6.30 ± 2.74	
Monthly income RMB	6,000 and below^a^	23.16 ± 6.87	**2.470^#^ (a** **>** **c)**	5.42 ± 3.65	**2.823^#^ (a** **>** **b)**	5.66 ± 3.79	1.492	6.14 ± 3.95	1.520	6.10 ± 2.60	1.294
	6,001 and above^b^	21.28 ± 6.39		3.72 ± 2.85		4.38 ± 3.08		4.83 ± 3.13		5.28 ± 2.17	
	I don't want to talk about^c^	19.23 ± 8.58		5.31 ± 2.98		5.23 ± 2.77		5.46 ± 2.96		6.23 ± 2.86	

*t = Student's t test, F = analysis of variance (ANOVA) test*.

# *p < 0.1*;

* *p < 0.05*;

** *p < 0.01*.

*Multiple comparisons between each two categories are done by post-hoc analysis (Fisher's least significant difference)*.

### Measurement Model Assessment

As outlined in Table 3, all indicator loadings range from 0.734 to 0.969 and are >0.70, as recommended by Hair et al. (59). All indicators are significant (*p* < 0.001) and confirm the indicator reliability. All Cronbach's alphas range from 0.806 to 0.928 and surpass the recommended threshold of 0.70 (59, 60). Furthermore, the composite reliability (CR) ranges from 0.886 to 0.941 and surpasses the suggested value of 0.70 (58, 60, 61). Thus, the internal consistency reliability is established.

**Table 3:** Measures of constructs reliability.

**Constructs/items**	**Standardized factor loading**	***t***	**CR**	**AVE**	**Cronbach's α**
Fear 1: I am most afraid of COVID-19.	0.737	16.371^***^	0.941	0.695	0.928
Fear 2: It makes me uncomfortable to think about COVID-19.	0.840	35.447^***^			
Fear 3: My hands become clammy when I think about COVID-19.	0.878	53.343^***^			
Fear 4: I am afraid of losing my life because of COVID-19.	0.734	16.012^***^			
Fear 5: When watching news and stories about COVID-19 on social media, I become nervous or anxious.	0.893	49.679^***^			
Fear 6: I cannot sleep because I worry about getting COVID-19.	0.866	35.991^***^			
Fear 7: My heart races or palpitates when I think about getting COVID-19.	0.873	40.134^***^			
Depression	0.862	41.745^***^	0.886	0.722	0.806
Anxiety	0.914	72.297^***^			
Stress	0.767	13.484^***^			
Turnover intention 1: Due to the impact of COVID-19, next year I will probably look for a new job outside this organization.	0.920	34.424^***^	0.937	0.832	0.898
Turnover intention 2: Lately, I have taken more interest in job-seeking websites due to COVID-19.	0.876	16.817^***^			
Turnover intention 3: Due to the current situation, I often think about quitting.	0.939	73.595^***^			

*COVID-19, coronavirus disease 2019; CR, composite reliability; AVE, average variance extracted*.

*** *p < 0.001*.

The values of average variance extracted (AVE) range between 0.695 and 0.832 and surpass the suggested value of 0.50 (58). These results confirm the convergent validity in all items.

Table 4 shows the cross-loadings of all items that ranked highest among the respective factors. Table 5 demonstrates that each construct of AVE is higher than the squared correlation between constructs. This corresponds with Fornell and Larcker's (61) recommendations. According to the findings, the measurement model is equally valid and acceptable and has discriminant validity, which means that analysis of the structural model can be performed.

**Table 4:** Cross-loadings of constructs.

**Scale items**	**Fear of COVID-19**	**Mental health**	**Turnover intention**
Fear 1	0.737	0.287	0.109
Fear 2	0.840	0.359	0.134
Fear 3	0.878	0.492	0.225
Fear 4	0.734	0.293	0.129
Fear 5	0.893	0.451	0.233
Fear 6	0.866	0.540	0.285
Fear 7	0.873	0.471	0.232
Depression	0.367	0.862	0.461
Anxiety	0.516	0.914	0.475
Stress	0.423	0.767	0.324
Turnover intention 1	0.231	0.467	0.920
Turnover intention 2	0.183	0.355	0.876
Turnover intention 3	0.248	0.518	0.939

*COVID-19, coronavirus disease 2019*.

**Table 5:** Correlations of constructs and AVE values.

**Construct**	**Mean ± SD**	**Fear of COVID-19**	**Mental health**	**Turnover intention**
Fear of COVID-19	22.54 ± 6.99	***0.695***		
Mental health	32.79 ± 21.18	0.274^**^	***0.722***	
Turnover intention	5.97 ± 2.57	0.050^**^	0.295^**^	***0.832***

*COVID-19, coronavirus disease 2019; AVE, average variance extracted*.

*Values on the diagonal in bold = AVE values; variables below the diagonal line = squared correlations between each pair of latent constructs*.

** *p < 0.01*.

### Structural Model Measurement

**Figure 2:**
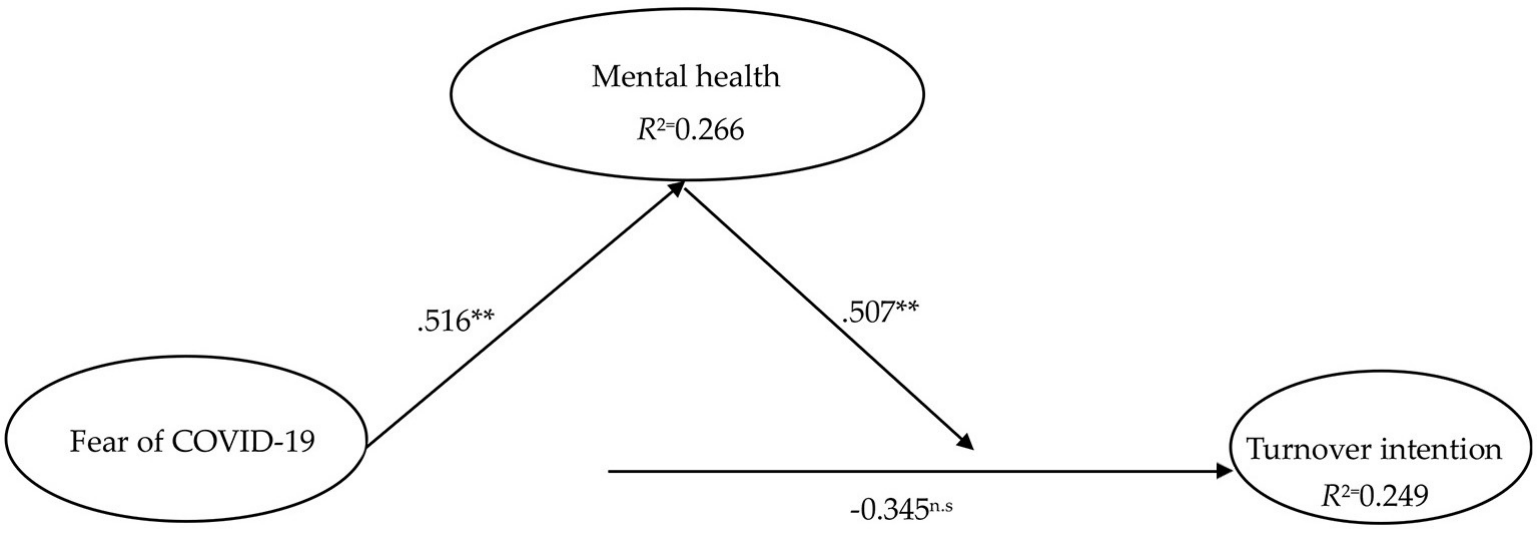
Partial least-squares (PLS) results. Source: own research. **p < 0.01; n.s., not significant.

The empirical results of the research model are displayed in Table 6 and Figure 2. With a sample size of 500, the bootstrap resampling method was applied to measure relationships within the theoretical model, produce *t*-values, and standard errors and to evaluate whether the path coefficient is significant.

**Table 6:** Structure model results.

**Hypotheses**	**Path**	**Path coefficient**	**Std. error**	***t*-value**	**Results**
Hypothesis 1	Fear → mental health	0.516	0.045	11.408^***^	Supported
Hypothesis 2	Mental health → turnover intention	0.507	0.074	6.897^***^	Supported
Hypothesis 3	Fear → turnover intention	−0.016	0.046	−0.345	Not supported

*** *p < 0.001*.

As the results show in Table 6, it is evident that fear of COVID-19 does not influence turnover intention (β = −0.016, *t* = −0.345, *p* > 0.05). The statistical results confirm that fear of COVID-19 positively influences depression, anxiety, and stress (β = 0.516, *t* = 11.408, *p* < 0.01). At the same time, depression, anxiety, and stress caused by the COVID-19 pandemic significantly positively impacts turnover intention (β = 0.507, *t* = 6.897, *p* < 0.01) and explains 24.9% of its variance. The connection paths among fear of COVID-19, mental health, and turnover intention are shown in Figure 2.

It should be noted that fear of COVID-19 alone does not influence turnover intention; however, depression, anxiety, and stress caused by fear of COVID-19 positively influences turnover intention.

## Discussion

This study investigates the impact of fear of COVID-19 on QH employees' mental health, specifically depression, anxiety, and stress and turnover intention. The main findings are as follows.

First, the mean score for fear of COVID-19 in QH employees in China exceeds the midpoint of 22.54 (SD: 6.99) and, when compared with similar studies, is higher than that of the population sample of Belarus (16.6) (62), Japan (18.71) (63), Russia, and Belarus combined (17.4) (64) and nurses in Pakistan (22.12) (22).

Second, the results reveal that QH employees report moderate-to-very severe symptoms of depression, anxiety, and stress: 43.4, 68.2, and 8.2%, respectively. The high prevalence of depression and anxiety could be attributed to increased exposure to guests suspected or confirmed to have COVID-19, apprehension over protecting themselves and their families from contracting COVID-19, and the increased workload of operating a QH.

Third, the QH employees in this study scored highly on the FCV-19S, which is associated with increased scores for their mental health status (specifically depression, anxiety, and stress). As there is a lack of studies involving QH employees, no direct comparison can be made. However, this outcome is consistent with previous studies that evidence that increased fear of COVID-19 is closely related to depression, anxiety, and stress (8, 26). While fear is believed to help motivate an individual to react effectively to a perceived threat, persistent, and extreme fear can cause adverse psychological responses, for example, depression, anxiety, and stress (65).

Fourth, increased scores in depression, anxiety, and stress are related to an increase in the turnover intention of QH employees. Depression, anxiety, and stress are the main psychological disorders that affect employees working in an organization. There were similar findings in literature on the hospitality sector, where depression caused employees to leave their jobs and seek employment elsewhere (47). Furthermore, another study evidences that intense anxiety and fear potentially negatively affect the health and well-being of nurses and how effectively they perform their duties during the pandemic (31).

Lastly, fear of COVID-19 does not directly influence the turnover intention of QH employees. This finding does not concur with the propositions of Barnett and Grabowski (52), De los Santos and Labrague (21), Mo et al. (66), and Ranney et al. (51), who all state that high levels of fear of COVID-19 increase turnover intention in nurses. This discrepancy may be due to differences in the study participants.

### Theoretical Contributions

The ongoing COVID-19 pandemic has adversely affected the global tourism industry (19, 67). This research provides a novel contribution toward understanding the negative effects fear of COVID-19 has on the mental health of QH employees. This study contributes to the psychological research of hospitality industry workers during the COVID-19 crisis in several ways.

First, as far as the authors' knowledge, this article is the first empirical survey investigating the associations among fear of COVID-19, mental health, and turnover intention among QH employees, thus providing key results in the field of hotel management.

Second, the mental health of QH employees is a major concern; however, it has rarely been discussed throughout the pandemic. This research applies DASS-21 to investigate the mental health status of QH employees in China. The empirical results confirm that depression, anxiety, and stress have a significant influence on turnover intention. These findings contribute to the extant knowledge base by uncovering the role of mental health in employee turnover intention. Thus, this research proposes that mental health should be considered an important factor in detecting employee turnover intention.

Finally, this study contributes to the literature on QH employee turnover intention. Throughout the COVID-19 pandemic, QHs provide accommodation, services, and meals for patients and medical/health-care workers. The QH also assists guests to comply with government quarantine orders by providing travelers and residents with accommodation for the mandatory 14-day quarantine period. QH employees face higher risks of infection than the general public and are affected not only physically by additional job requirements but also psychologically by the increased threat of contracting COVID-19. Research on QH employees is sparse; thus, it is necessary to investigate the role of QH employees in the field of hotel management.

### Practical Implications

Resilience and sustainability in the hospitality industry can help businesses recover from a public health crisis by strengthening hotel leadership (68). As excessive fear may exacerbate existing mental health problems and ultimately affect the health and turnover intention of QH employees, hoteliers should give priority to supporting the mental health of their staff during a crisis. Under these circumstances, the following implications are provided for managers of QHs.

First, in order to decrease fear of COVID-19, the QH managers should implement a well-planned workplace protocol, such as relevant training, response plans, and guidelines and safety practices for caring for quarantine guests. Training is a crucial element to the readiness and competence of QH employees when faced with disease outbreaks or other forms of disaster. To ensure safe and smooth operation of the adjusted QH, employees must be knowledgeable, skilled, oriented, and proficient in all QH protocols.

Second, this study's results evidence that QH employees have moderate-to-very severe mental health issues, including symptoms of depression, anxiety, and stress, which will negatively influence turnover intention. Based on this finding, it is suggested that QH managers build an open communication plan to mitigate uncertainty and decrease the anxiety levels of QH employees. Open communication pathways provide employees with clear information and updates on the pandemic, the status of quarantine guests, changes to policies and procedures, and impacts to workloads. It also encourages employees to communicate their demands and concerns, which is a significant resource for promoting resilience.

Third, QH managers should cultivate an adequate psychological support program to mitigate and prevent symptoms of declining mental health. For example, periodical online surveys could be used to evaluate and observe the mental health of employees. If any negative psychological symptoms are identified, immediate psychological support, and an appropriate amount of time away from work should be provided. At the same time, hoteliers should develop online mental health education materials and counseling to provide psychological assistance.

Exceptional employee performance is a precious asset for hoteliers. By reducing the mental health issues of employees working in QHs, turnover intention will also decline; and hopefully, after the pandemic is pacified, employees will want to contribute toward the hotel's recovery performance.

Fourth, to reduce the risk of transmitting COVID-19 and cross-contamination, it is suggested that hoteliers implement technological solutions, such as robotic room service to supply meals and a zero-contact check-out. The provision of contactless services is as an effective management tool, as reducing contact with quarantine guests and minimizing their time in public areas will reduce the risk of transmitting COVID-19. The rapid development of non-contact and automated check-in/out processes is essential for the hotel industry both mid- and post-pandemic (69, 70).

Lastly, hoteliers should also comply with additional benefits strategies, such as reducing workloads or hours, to ensure employee job satisfaction and reduce any psychological impact.

This study is designed to aid hoteliers by highlighting the rectifiable issues and assist the industry's recovery post-pandemic. The findings identify the prospective suable issues in order to facilitate the development of human resource management toward supporting employees' psychological health within the hospitality industry.

## Limitations and Future Research Directions

As the convenience sampling strategy was used and the only study samples collected were of QH employees in Xiamen, China, the representativeness of the results is limited. In order to improve the representativeness and generality of sampling, it is necessary to adopt a more systematic and inclusive sampling method. Researchers are also encouraged to explore QH employees in different countries, and it is recommended that future studies focus on sampling employees in different departments, such as reception, housekeeping, and room service. The use of different data collection methods would also be beneficial. Even though this study used the structural equation model, the research results did not confirm any specific causal relationships between constructs.

## Data Availability Statement

The raw data supporting the conclusions of this article will be made available by the authors, without undue reservation.

## Ethics Statement

The study protocol was reviewed and approved by the Executive Principal of Yango University, China. An oral consent that follows the principles in the Declaration of Helsinki was obtained from all study participants, and they were instructed to participate or withdraw from the study at any stage voluntarily. Anonymized data were used for analysis and interpretation.

## Author Contributions

All authors listed have made a substantial, direct and intellectual contribution to the work, and approved it for publication.

## Conflict of Interest

The authors declare that the research was conducted in the absence of any commercial or financial relationships that could be construed as a potential conflict of interest.
